# Alternative oxidase encoded by sequence-optimized and chemically-modified RNA transfected into mammalian cells is catalytically active

**DOI:** 10.1038/s41434-021-00235-z

**Published:** 2021-03-04

**Authors:** Luca Giordano, Manish K. Aneja, Natascha Sommer, Nasim Alebrahimdehkordi, Alireza Seraji, Norbert Weissmann, Carsten Rudolph, Christian Plank, Howard T. Jacobs, Marten Szibor

**Affiliations:** 1 Faculty of Medicine and Health Technology, FI-33014 Tampere University, Tampere, Finland; 2 Institute of Biotechnology, FI-00014 University of Helsinki, Helsinki, Finland; 3 Excellence Cluster Cardio-Pulmonary Institute (CPI), University of Giessen and Marburg Lung Center (UGMLC), Member of the German Center for Lung Research (DZL), Justus-Liebig University Giessen, D-35392 Giessen, Germany; 4 Ethris GmbH, DE-82152 Planegg, Germany; 5 Department of Pediatrics, Ludwig Maximilian University of Munich, DE-80337 Munich, Germany; 6 Institute of Molecular Immunology and Experimental Oncology, Klinikum rechts der Isar, Technical University of Munich, DE-81675 Munich, Germany; 7 Department of Cardiothoracic Surgery, Jena University Hospital, DE-07747 Jena, Germany; 8 Present Address: School of Medicine, Division of Cardiology, Center for Metabolism and Mitochondrial Medicine, and Vascular Medicine Institute, University of Pittsburgh, Pittsburgh, PA 15261 USA

**Keywords:** Nucleic-acid therapeutics, Transcription, Transfection

## Abstract

Plants and other organisms, but not insects or vertebrates, express the auxiliary respiratory enzyme alternative oxidase (AOX) that bypasses mitochondrial respiratory complexes III and/or IV when impaired. Persistent expression of AOX from *Ciona intestinalis* in mammalian models has previously been shown to be effective in alleviating some metabolic stresses produced by respiratory chain inhibition while exacerbating others. This implies that chronic AOX expression may modify or disrupt metabolic signaling processes necessary to orchestrate adaptive remodeling, suggesting that its potential therapeutic use may be confined to acute pathologies, where a single course of treatment would suffice. One possible route for administering AOX transiently is AOX-encoding nucleic acid constructs. Here we demonstrate that AOX-encoding chemically-modified RNA (cmRNA), sequence-optimized for expression in mammalian cells, was able to support AOX expression in immortalized mouse embryonic fibroblasts (iMEFs), human lung carcinoma cells (A549) and primary mouse pulmonary arterial smooth muscle cells (PASMCs). AOX protein was detectable as early as 3 h after transfection, had a half-life of ~4 days and was catalytically active, thus supporting respiration and protecting against respiratory inhibition. Our data demonstrate that AOX-encoding cmRNA optimized for use in mammalian cells represents a viable route to investigate and possibly treat mitochondrial respiratory disorders.

## Introduction

The mitochondrial electron transport chain (ETC) is a central component of cellular metabolism, consisting of four respiratory complexes (cI-cIV) that link proton translocation across the inner mitochondrial membrane to the step-wise transfer of electrons. Impaired electron flux in the ETC therefore affects both upstream and downstream processes such as the tricarboxylic acid cycle and mitochondrial ATP production, respectively. In consequence, ETC deficiencies are the underlying cause of a large number of severe metabolic disorders [1, 2], which currently lack effective therapies [3].

Nevertheless, naturally evolved by-pass mechanisms exist in plants and lower organisms that can overcome ETC inhibition. Alternative respiratory chain enzymes have two main representatives. First, there are alternative dehydrogenases (e.g., NADH dehydrogenase from yeast, Ndi1), which transfer electrons from NADH to ubiquinone thereby generating its reduced form ubiquinol. Second, there are alternative oxidases (AOX), which complete the electron transfer from ubiquinol to oxygen [4, 5]. Here we used AOX from *Ciona intestinalis*, a di-iron protein, that like all alternative enzymes acts in a non-proton-motive reaction [6, 7]. Notably, AOX by-passes two proton pumps of the ETC, namely cIII and cIV [6, 7], and represents a metabolic rescue mechanism that is absent in vertebrates [3]. Notwithstanding its natural absence, AOX can be xenotopically expressed in a catalytically active form in human cells with primary respiratory chain dysfunction [8] or mouse mtDNA-depleted cells [9], in drosophila disease models [10–15], and in the mouse [7, 16] by using an AOX cDNA transiently expressed or integrated into specific sites in the genome. Remarkably, in most cases this has been achieved without producing adverse phenotypic effects, at least under standard physiological conditions, i.e., in the absence of metabolic stress signaling related to respiratory disruption [7]. By enabling electron flow through cI and other ubiquinone reductases, AOX can maintain the mitochondrial membrane potential, thus supporting ATP production, and blunt the excessive production of reactive oxygen species when the respiratory chain is impaired [6, 17]. Furthermore, AOX expressed in mouse was shown to alleviate the acute lethal effects of cyanide administration [7] and of LPS endotoxemia [18], attenuate cigarette smoke-induced lung damage [19], and block hypoxia-induced pulmonary vasoconstriction in an ex vivo setting [20], illustrating its potential for use in future therapy against diseases associated with ETC dysfunction. These data contrast with findings in other mouse models of respiratory disruption in which persistent AOX expression exacerbated pathological features of mitochondrial myopathy [21] and promoted maladaptive remodeling upon cardiac ischemia-reperfusion [17]. This suggests that chronic AOX expression may be disadvantageous if it disrupts intracellular and/or intercellular signaling processes required to mount a response to specific pathological insults. These drawbacks will need to be considered in any future development of AOX as a therapy, and suggest that its usefulness may be confined to acute pathologies, where a single course of treatment would suffice, or to cases where it can be targeted to specific organs or tissues.

Possible routes for administering AOX therapeutically include AOX protein or AOX-encoding nucleic acid constructs. The former is inherently problematic, due to its likely immunogenicity [22, 23], as well as the predicted hydrophobicity of the AOX protein [24], despite recent advances in solubilizing membrane proteins [25]. Gene transfer using DNA constructs, on the other hand, carries the risk of unintended integration into the genome, with potentially mutagenic/oncogenic consequences [26], even for a theoretically non-integrating vector, such as adeno-associated virus [27]. Delivery of RNA-based therapeutics has therefore emerged as a viable alternative, particularly since the use of chemically modified RNA (cmRNA) [28] potentially negates major disadvantages such as RNase-mediated decay [29] and activation of the innate immune response via Toll-like receptors [30]. Furthermore, cmRNA was demonstrated to be stable and essentially non-immunogenic even if administered repeatedly [31]. Efficient targeting of the gene product to mitochondria should additionally minimize any detrimental metabolic or immune response although mitochondrially derived antigens may be presented at the cell surface, e.g., by MHC class I molecules. Such an event, however, has thus far only been described for pathological conditions in which the Pink1-Parkin system is disturbed [32].

In the present study, we therefore set out to test whether an AOX-encoding cmRNA, codon-optimized for use in mammalian cells, and the introduction of alternative targeting information would suffice to produce an enzymatically active protein. Such a validation is a prerequisite for any future therapeutic use in vivo.

## Materials and methods

### Design and generation of cmRNA constructs

AOX-encoding constructs engineered for cmRNA production were custom-synthesized by GenScript (Piscataway, NJ). The GeneOptimizer software (GeneArt) [33] was applied for codon-optimization of the coding sequences, i.e., mitochondrial targeting sequence, MTS, and alternative oxidase, AOX, for use in humans. For cmRNA production, procedures for transcription and transcript maturation were applied as previously described (‘modification 2’) [34]. For the sake of clarity, 35% iodouridine and 65% unmodified uridine, 7.5% iodocytidine and 92.5% unmodified cytidine together with unmodified adenine and guanine were incorporated into the in vitro transcription reaction. cmRNA was further polyadenylated by using a poly(A) polymerase. Poly(A) length was determined by capillary gel electrophoresis to be between 200 and 300 nucleotides. Annotated information on cmRNA sequences is available from GenBank (cmRNA-ATP5F1B-AOX [MW222236], cmRNA-ATP5F1B-mutAOX [MW222237], cmRNA-HBA1-AOX [MW222238], cmRNA-HBA1-mutAOX [MW222239], cmRNA-minimal-AOX [MW222240], cmRNA-minimal-mutAOX [MW222241]).

### Cell isolation and cell culture conditions

Primary mouse embryonic fibroblasts (MEFs) were isolated from wild-type (WT) and AOX-transgenic (AOX^Rosa26^) [7] embryos (E13.5–15.5) in the C57BL/6 background and immortalized (generating iMEFs) as described elsewhere [35, 36]. MEFs, iMEFs, and human lung carcinoma (A549) cells (86012804, Sigma-Aldrich/Merck Life Science) were grown in Dulbecco Modified Eagle’s Medium (DMEM) supplemented with 4.5 g/l glucose (BE12-614F, Lonza/BioNordika), 10% fetal bovine serum (FBS, 10270106, Gibco/ThermoFisher Scientific), 100 U/ml penicillin plus 100 μg/ml streptomycin (P0781, Sigma-Aldrich/Merck Life Science), and 2 mM L-glutamine (BE17-605E, Lonza/BioNordika). Mouse pulmonary artery smooth muscle cells (PASMCs) were isolated from precapillary pulmonary arterial vessels of WT C57Bl/6 mice as previously described [37]. PASMCs were cultured in Smooth Muscle Cell Growth Medium (C-22062, PromoCell) supplemented with 10% FCS (F7524, Sigma-Aldrich/Merck Life Science) and 0.002% Normocin (ant-nr-1, InvivoGen).

### Cell transfection

2.5 × 10^5^ iMEFs or A549, or 6 × 10^5^ PASMCs were used for transfection using Lipofectamine 2000 Transfection Reagent (11668019, ThermoFisher Scientific) following the manufacturer’s instructions in growth medium without antibiotics. To test for transfection efficiency, we measured fluorescence intensities of upon cell transfection in 4–5 areas using CellProfiler, an open-source software for quantitative analysis of biological images. This revealed approximately 36% AOX positive cells/total number of cells (AOX/Hoechst) when using 0.6 µg of AOX cmRNA/ml cell culture medium 24 h after transfection.

### Mitomycin C treatment

5 × 10^6^ MEFs were plated in a T175 flask (660175, Greiner) and cultured overnight, then incubated for 3 h with 10 µg/ml mitomycin C (M4287, Sigma-Aldrich/Merck Life Science) in DMEM-glucose, without FBS and antibiotics. Mitomycin C was washed out and cells were detached with trypsin. After counting, 7.5 × 10^5^ mitomycin C-treated cells were plated in a T25 flask (661160, Greiner) and cultured overnight. Finally, cells were transfected with AOX cmRNA for 24 h, then grown in fresh media and collected every day over a period of 7 days.

### Protein extraction and Western blotting

Protein extraction, SDS-PAGE and protein transfer were carried out as previous described [19]. Membranes were reacted overnight with the following primary antibodies: custom-raised rabbit anti-AOX (1:40,000, 21st Century Biochemicals) [8], mouse anti-Total OXPHOS Rodent WB Antibody Cocktail (1:250, ab110413, Abcam), mouse anti-α-smooth muscle actin (anti-α-SMA, 1:8,000, A2547, Sigma-Aldrich/Merck Life Science), anti-α-tubulin (1:2,000, 3873, Cell Signaling). After washing with TBST, membranes were incubated for one hour at room temperature with the secondary antibodies: Peroxidase-AffiniPure goat Anti-Mouse IgG (1:10,000, 115-035-003, Jackson ImmunoResearch), Peroxidase-AffiniPure goat Anti-Rabbit IgG (1:20,000, 111-035-144, Jackson ImmunoResearch). Blots were developed using Clarity Western ECL Substrate (1705061, Bio-Rad), and detected by a ChemiDoc Imaging System (Bio-Rad). Quantitative analyses were performed using Image Lab software (Bio-Rad).

### Immunocytochemistry

Following transfection, iMEFs, cultured for 24 h on 35 mm Nunc glass bottom dishes (ThermoFisher Scientific), were fixed using 2% paraformaldehyde (158127, Sigma-Aldrich/Merck Life Science) for 20 min at 4 °C, permeabilized using 0.3% Triton X-100 (93443, Sigma-Aldrich/Merck Life Science) in 2% paraformaldehyde for 20 min, and blocked using 5% BSA for 30 min at room temperature. Cells were stained with primary antibodies diluted in 5% BSA overnight at 4 °C as follows: custom-raised rabbit anti-AOX (1:40,000, 21st Century Biochemicals) [8] and mouse anti-ATP5A (1:500, ab14748, Abcam). After rinsing for three times in PBS for 10 min, cells were probed for one hour at room temperature with the following secondary antibodies: Alexa Fluor 488 goat anti-rabbit (1:2000 in 0.3% Triton X-100, A-11008, ThermoFisher Scientific) and Alexa Fluor 594 goat anti-mouse (1:2000 in 0.3% Triton X-100, A-11005, ThermoFisher Scientific) diluted in 0.3% Triton X-100 in PBS, followed by three 10 min washes in PBS. Nuclei were stained using 0.15 µg/ml Hoechst (H1399, ThermoFisher Scientific). Images were acquired using a Leica TCS SP5 II confocal microscope (Leica Microsystems), displayed as maximum z-projections and adjusted using Fiji ImageJ imaging software.

### High-resolution respirometry

Whole-cell and permeabilized cell respiration was measured as described previously [38] with minor modifications. Briefly, 5 × 10^6^ iMEFs or A549 cells were plated in a T175 flask (660175, Greiner), or 6.5 × 10^5^ mouse PASMCs in a 10 cm dish (664160, Greiner), and transfected 24 h before the experiments. Growth medium was replaced 1 h before the assay. Cells were detached with 0.05% trypsin and counted by trypan blue exclusion. After centrifugation at 200 *g*_max_ for 5 min at 25 °C, medium was removed and cells were suspended in PBS and aliquoted for respirometry and protein extraction. Mitochondrial respiration was assayed using an O2k oxygraph (Oroboros Instruments, Innsbruck, Austria). 2 × 10^6^ iMEFs or A549 cells, or 1 × 10^6^ PASMCs were directly resuspended in the oxygraph chamber in 2 ml of respiration medium B [19, 38]. After measuring endogenous whole-cell respiration, cells were permeabilized by the addition of digitonin (30–50 μg) and substrates and inhibitors were added in the following order and final concentrations: sodium pyruvate (5 mM), sodium glutamate (5 mM) and sodium malate (2 mM), ADP (2 mM), rotenone (0.5 µM), sodium succinate (10 mM), antimycin A (2.5 µM), n-propyl gallate (nPG, 200 μM), N,N,N′,N′-tetramethyl-phenylenediamine (1 mM) plus sodium L-ascorbate (2 mM), sodium azide (40 mM). Oxygen consumption was normalized to the total protein content measured by the Bradford assay. Chemicals were purchased from Sigma-Aldrich/Merck Life Science.

### Antimycin A cytotoxicity assay

2.5 × 10^5^ iMEFs were plated in a six-well plate (657160, Greiner), transfected with cmRNA for 24 h, and incubated for 48 h in DMEM lacking glucose, glutamine and phenol red (A14430, Thermo Fisher) supplemented with 10 mM galactose (G0750, Sigma-Aldrich/Merck Life Science), 10% FBS (12070, Gibco), 100 U/ml penicillin and 100 μg/ml streptomycin (DE17-602, Lonza), 2 mM L-glutamine (BE17-605E, Lonza), 1 mM sodium pyruvate (S8336, Sigma-Aldrich/Merck Life Science), and 5 µM antimycin A (8674, Sigma-Aldrich/Merck Life Science). The numbers of total and dead cells were counted using a hematocytometer by trypan blue exclusion. Representative images per well were acquired using an Axio Vert.A1 inverted microscope, processed by ZEN 2 imaging software (Zeiss) and color-inverted (white, black) by Fiji ImageJ software.

### Statistical analyses

All data are shown as mean ± standard deviation. To ensure adequate power and reproducibility, all experiments were replicated a minimum of three times, except where stated in figure legends. The number of samples per experiment was limited by the number that could be conveniently co-analyzed, such as on a single gel or in a single respirometry or microscopy session. No samples were excluded. Samples were not randomized. The variance was similar between the groups that have been statistically compared. A *p* value ≤ 0.05 was considered statistically significant. Data were analyzed using GraphPad Prism software (v8, GraphPad Software) with analyses and post hoc tests as indicated.

### Image manipulation

Blot and immunocytochemistry images were adjusted for brightness and contrast, cropped, resized and annotated for presentation, but not manipulated in any other way.

## Results and discussion

### Design of chemically-modified AOX RNA (cmRNA)

**Fig. 1:**
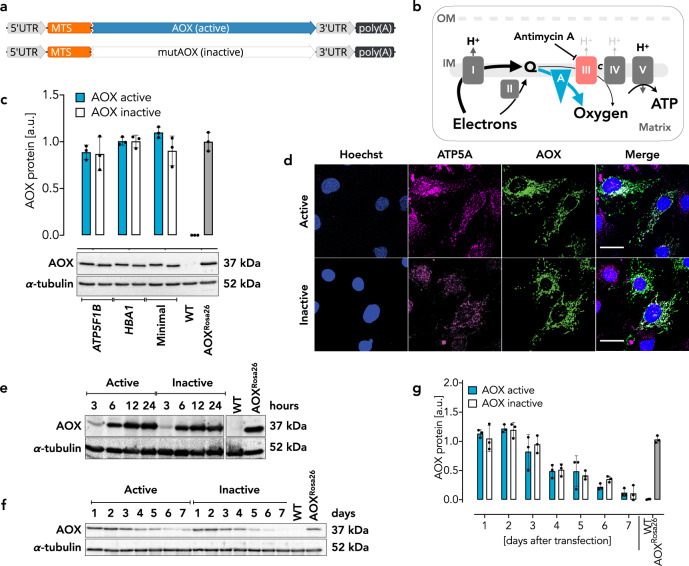
Construct design and expression test in iMEFs, of different AOX cmRNA variants. **a** Schematic of cmRNA design. AOX, codon-optimized *Ciona intestinalis* alternative oxidase; mutAOX, catalytically inactive form of codon-optimized AOX; UTR, untranslated region; MTS, human ATP5F1B-derived mitochondrial targeting sequence; poly(A), polyadenylate tail. **b** Schematic illustrating where AOX integrates into the mitochondrial respiratory chain of mammalian cells and how AOX branches off electrons from ubiquinol to reduce oxygen to water. I–V, respiratory chain complexes. A, alternative oxidase (AOX). *c*, cytochrome *c*. IM, inner mitochondrial membrane. OM, outer mitochondrial membrane. Q, quinone pool. **c** Representative Western blots of iMEF lysates 24 h after transfection with catalytically active or inactive cmRNA constructs as indicated. α-tubulin, loading control. “ATP5F1B”, “HBA1” and “Minimal” indicate the different 5′ UTR constructs used. WT (wild-type) and AOX^Rosa26^ (AOX-transgenic) iMEFs serving as negative and positive control for AOX expression, respectively. **d** Immunocytochemistry of iMEFs transfected with AOX cmRNA constructs using human ATP51B 5’UTR. Hoechst, nuclear stain; ATP5A, mitochondrial stain. Scale bars, 30 µm. **e** Representative Western blots using iMEFs transfected with cmRNA constructs bearing ATP51B 5′ UTR and encoding catalytically active or inactive AOX as indicated. Time after transfection shown in hours. α-tubulin, loading control. **f** Representative Western blots of MEFs, pre-treated with mitomycin C, transfected with cmRNA constructs as indicated. Time after transfection shown in days. α-tubulin, loading control. **g** Densitometric analysis of AOX protein expression in cmRNA-transfected MEFs normalized to α-tubulin. All data are shown as mean ± SD in arbitrary units (a.u.) with one being the average expression seen in AOX^Rosa26^ iMEFs or MEFs of *n* = 3 independent experiments.

In order to design a possible AOX therapeutic, we first deleted from the *Ciona intestinalis* AOX cDNA the native mitochondrial targeting sequence (MTS, AOX amino acids 1–51, as predicted by MitoProt II–v1.101 [39]), so as to avoid the potential risk that, after mitochondrial import and cleavage, the released MTS peptide might evoke an unwanted stress or immune response yet allowing proper integration into the respiratory chain (Fig. 1a,b). In its place, we fused a substitute MTS, derived from human ATP synthase F1 subunit beta (ATP5F1B, NCBI human gene ID: 506, amino acids 1–63) to the N-terminus of the truncated AOX sequence (AOX amino acids 52–369) (Fig. 1a). To be able to distinguish AOX-mediated effects from transfection-related artefacts, we generated construct pairs for this and for subsequent variants, respectively encoding either the catalytically active AOX or a catalytically inactive mutant thereof, previously described [40]. We next used MitoProt II [39] to predict the probability of mitochondrial targeting of the AOX fusion proteins and obtained 0.9535 for the catalytically active form and 0.9611 for its catalytically inactive counterpart. To generate cmRNA transcripts with putatively high stability and low immunogenicity, different modifications and codon-optimizations were applied that have been described previously for non-mitochondrial proteins (“modification 2”) [34]. Finally, transcripts were flanked by regulatory (untranslated) regions (UTRs) (Fig. 1a) to guarantee proper translation initiation, transcript stabilization and subcellular localization [41, 42]. All constructs contained 163 nt (nucleotides) of the human ATP5F1B 3′ UTR, which has been reported to target mRNA to ribosomes located in the vicinity of mitochondria [43]. Three 5′ UTR constructs were generated: a minimal sequence comprising 14 nt of the T7 promoter combined with a Kozak consensus sequence [34]; a 102 nt element from the human ATP5F1B 5′UTR, or 30 nt derived from the human hemoglobin alpha 1 subunit (HBA1, NCBI human gene ID: 3039) 5′ UTR (Fig. 1a).

### AOX cmRNAs are rapidly translated into stable protein in iMEFs

To evaluate the stability and utility of the designed cmRNA constructs we tested them in different mammalian cells. We first transfected iMEFs with each of the three AOX cmRNA pairs (catalytically active and inactive versions of each, 0.6 µg cmRNA per ml of growth medium). Semiquantitative analysis by Western blot revealed that transfected iMEFs showed AOX protein expression levels similar to those seen in iMEFs derived from AOX transgenic mice (AOX^Rosa26^) [7]. Importantly, the different 5′ UTRs had no impact on the overall efficiency of cmRNA translation (Fig. 1a,c). We also tested the subcellular localization of AOX by immunocytochemistry, 24 h after transfection. AOX staining showed a characteristic mitochondrial network pattern, overlapping the distribution of ATP5A, a mitochondrial marker protein (Fig. 1d and Supplementary Fig. S1). Overall expression and subcellular targeting to mitochondria were not prevented by codon optimization and MTS substitution, and were also essentially unaffected by catalytic activity or 5′ UTR variation. In the following experiments we therefore used the individual construct pairs interchangeably.

Considering a potential use of AOX cmRNA species specifically in the rapid treatment of acute pathologies, the earliest detection of AOX protein is of great importance. Western blot analysis of iMEFs transfected with cmRNA using the ATP5F1B 5′ UTR revealed detectable AOX protein as early as 3 h after transfection, reaching a plateau level by ~12 h (Fig. 1e). Equally important for possible therapeutic use is to estimate the persistence of expression which should depend on the stability of both the cmRNA and the protein. To measure AOX protein half-life following transfection with the ATP5F1B 5′ UTR cmRNA, we used non-immortalized mouse embryonic fibroblasts (MEFs) that had been treated for 3 h prior to transfection with 10 μg/ml mitomycin C, a DNA alkylating agent impairing DNA synthesis and transcription, and therefore cell proliferation [44]. Mitomycin C thus transforms proliferative cells into a post mitotic differentiation state and thereby allows the estimation of true AOX protein decay instead of “dilution” effects due to cell proliferation. AOX protein levels were quantified by Western blot over a time course of 7 days (Fig. 1f) revealing a half-life for AOX protein expressed by cmRNA of ~4 days irrespective of its catalytic activity (Fig. 1g).

### cmRNA-encoded AOX expressed in iMEFs is catalytically active

Using respirometry, we next tested whether the cmRNA-encoded AOX expressed in iMEFs is catalytically functional. In trial experiments, we transfected iMEFs with increasing amounts of AOX cmRNA and tested both oxygen consumption of digitonin-treated cells (Supplementary Fig. S2a) as well as protein expression (Supplementary Fig. S2b). AOX-specific respiration was estimated by subtracting the residual oxygen consumption in the presence of the AOX inhibitor nPG from that in the presence of the cIII inhibitor antimycin A, using a cII-linked substrate (succinate), while inhibiting cI respiration with rotenone. It may also be noted that, across an 8-fold concentration range of cmRNA (Supplementary Fig. S2a), there was no substantial impairment to AOX-mediated respiration resulting from over-expression. Based on these data, we implemented a more extensive experiment using iMEFs transfected with batches of 1.2 µg cmRNA per ml of growth medium, using the various constructs.

**Fig. 2:**
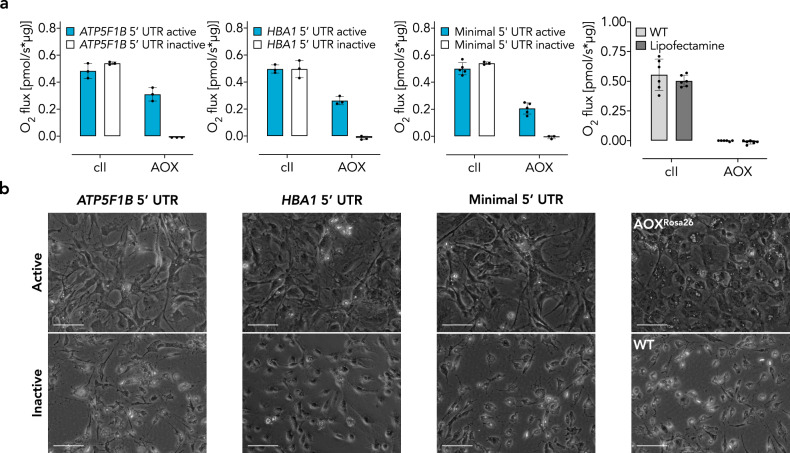
cmRNA-encoded AOX is catalytically active in iMEFs. **a** Respirometry using permeabilized iMEFs transfected with AOX-encoding cmRNA variants as indicated. WT iMEFs treated with or without lipofectamine served as negative control. Date are shown as mean ± SD of *n* ≥ 3 experiments. cII, respiratory complex II activity upon addition of succinate in the presence of cI inhibitor rotenone; AOX, *Ciona intestinalis* alternative oxidase activity upon further addition of antimycin A normalized to AOX inhibitor n-propyl gallate (nPG). **b** Representative images showing cellular morphology of iMEFs transfected with AOX cmRNA variants as indicated and treated with antimycin A (5 µM) for 48 h in 10 mM galactose-containing medium. Scale bars indicate 30 µm.

Transfection with each of the constructs had no effect on respiration via the standard ETC, whereas oxygen consumption was resistant to antimycin A only after transfection with those predicted to be catalytically active (Fig. 2a). We also performed a cytotoxicity assay to test for functional AOX activity in cultured iMEFs. To enforce oxidative metabolism [45], we incubated the transfected iMEFs in galactose-containing medium. Only iMEFs expressing a catalytically active form of AOX (independent of the cmRNA design) survived a lethal dose of antimycin A under such conditions (Fig. 2b, Supplementary Fig. S2c, d).

### cmRNA-encoded AOX is expressible and active in other cell types

**Fig. 3:**
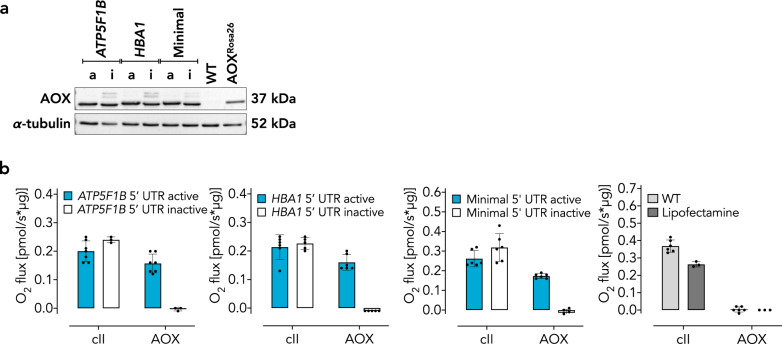
cmRNA-encoded AOX is catalytically active in A549 cells. **a** Representative Western blots of A549 cells 24 h after transfection with AOX cmRNAs as indicated (nomenclature as in Fig. 1; a, active; i, inactive). **b** Respirometry using A549 cells transfected with AOX cmRNA variants. Nomenclature as in Fig. 2. Data are shown as mean ± SD of values from *n* > 3 experiments, shown individually as filled circles.

**Fig. 4:**
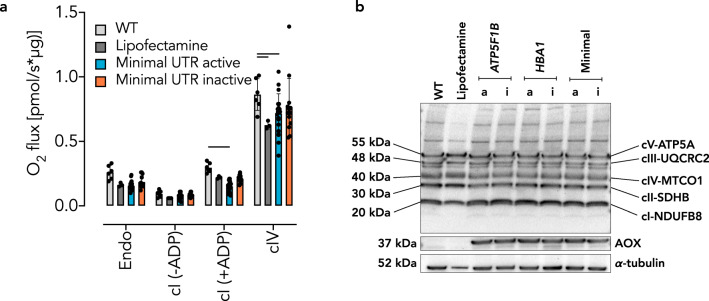
AOX cmRNA transfection has minor effects on A549 cell respiration and expression of respiratory subunits. **a** Respirometry of A549 cells transfected with AOX cmRNAs encoding active or inactive AOX as indicated. Endo, oxygen consumption of intact cells prior to permeabilization with digitonin (endogenous substrates); cI (−ADP), measurements made after permeabilization and supplementation with complex I-linked substrates (pyruvate 5 mM, glutamate 5 mM, malate 2 mM) in absence of ADP (non-phosphorylating respiration); cI (+ADP), measurements made after the further addition of ADP (phosphorylating respiration, state 3); cIV, measurements in the presence of antimycin A and after the addition of ascorbate/TMPD and the subtraction of residual oxygen consumption (resistant to azide). Data are shown as mean ± SD of *n* ≥ 3 experiments (indicated individually by filled circles), with horizontal bars representing significant differences (*p* ≤ 0.05) based on two-way ANOVA and Tukey’s multiple comparisons test. **b** Representative Western blots (*n* = 2) probed for selected subunits of the mitochondrial respiratory complexes as indicated, as well as AOX, and α-tubulin used for loading control.

**Fig. 5:**
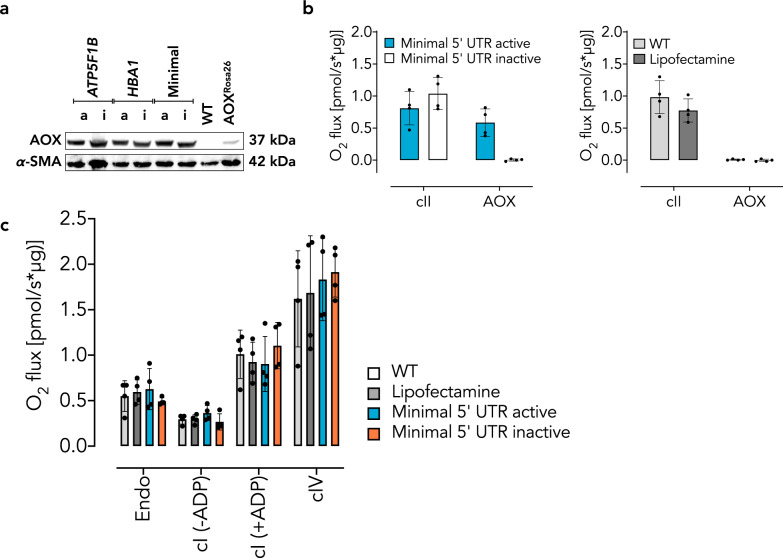
cmRNA-encoded AOX is expressed and catalytically active in primary mouse pulmonary artery smooth muscle cells (PASMCs). **a** Representative Western blots (*n* = 2) and **b** respirometry, using the same nomenclature as Fig. 4, except that the loading control used in the Westerns was α-SMA, as indicated. Data are shown as mean ± SD of values from *n* = 4 experiments, indicated individually by filled circles. **c** Respirometry using the same nomenclature as Fig. 4. Data are shown as mean ± SD of values from *n* = 4 experiments, indicated individually by filled circles.

We recently demonstrated an essential role for mitochondrial respiratory chain function in acute pulmonary oxygen sensing [20]. Furthermore, xenotopic expression of AOX attenuated lung tissue destruction and loss of function in mice chronically exposed to cigarette smoke [19], and prevented acute oxygen sensing in PASMCs [20]. We thus conducted experiments using A549 cells, a cell line originally derived from type II alveolar epithelial cells. 24 h after transfection with cmRNA, we found robust AOX expression in A549 cells for each of the variants (Fig. 3a) as well as enzymatic activity where expected (Fig. 3b). In A549 cells cmRNA-encoded AOX did not affect endogenous cellular respiration (Fig. 4a). Equally, cI-driven or cIV-driven respiration in permeabilized A549 cells was unaffected by cmRNA-encoded AOX, in comparison with controls using only the lipofectamine transfection reagent (Fig. 4a). Furthermore, Western blot analysis revealed that cmRNA-encoded AOX did not alter the expression of selected subunits of the respiratory chain complexes (Fig. 4b). Similar results were obtained with PASMCs (Fig. 5a–c).

## Conclusion

In this study we successfully generated cmRNAs coding for a humanized version of AOX, which were introduced into three different human and mouse cell-lines by lipofection. AOX cmRNA constructs incorporating different 5′ UTR elements were expressed at comparable levels, and expression persisted for at least six days in iMEFs that were prevented from proliferating, without evident off-target effects. The expressed protein exhibited enzymatic activity, whilst a version engineered to abolish such activity did not.

AOX has been postulated as a therapeutic agent for use in diseases characterized by mitochondrial dysfunction. The characterized AOX cmRNA constructs should now ideally be tested in a collection of cell-lines with primary respiratory chain defects, as have been used previously to demonstrate the effects of conventional transfection using plasmids and lentivectors [8]. Following such trials, in vivo tests in suitable models would be justified. Notably, long-term (or even transgenic) expression may cause maladaptive organ remodeling [17, 21] and possibly should be use with caution in patients with a history of neoplastic disease [9]. In our previous studies, we highlighted potential advantages of using AOX in acute pathologies such as cyanide (or cigarette smoke) intoxication [7, 19] and endotoxemia [18] as well as acute pulmonary oxygen sensing [20]. The rapid expression and biochemical properties of the cmRNA constructs make an application under such acute conditions a viable option. Furthermore, there is no obvious way of safely delivering AOX to affected tissues as a protein without eliciting an immune reaction; nor is there any certainty of avoiding the risks of insertional mutagenesis when introducing it in DNA form. Using cmRNA as a vehicle avoids both problems. Our findings here indicate that this approach can indeed be successful. It will be crucial to test the ability to introduce AOX in vivo, at the whole organism level, and establish the limits of its efficacy in appropriate disease models.

### Supplementary information

Supplemental Material
