# Correction: Alternative oxidase encoded by sequence-optimized and chemically-modified RNA transfected into mammalian cells is catalytically active

**DOI:** 10.1038/s41434-024-00473-x

**Published:** 2024-08-15

**Authors:** Luca Giordano, Manish K. Aneja, Natascha Sommer, Nasim Alebrahimdehkordi, Alireza Seraji, Norbert Weissmann, Carsten Rudolph, Christian Plank, Howard T. Jacobs, Marten Szibor

**Affiliations:** 1 Faculty of Medicine and Health Technology, FI-33014 Tampere University, Tampere, Finland; 2 Institute of Biotechnology, FI-00014 University of Helsinki, Helsinki, Finland; 3 Excellence Cluster Cardio-Pulmonary Institute (CPI), University of Giessen and Marburg Lung Center (UGMLC), Member of the German Center for Lung Research (DZL), Justus-Liebig University Giessen, D-35392 Giessen, Germany; 4 Ethris GmbH, DE-82152 Planegg, Germany; 5 Department of Pediatrics, Ludwig Maximilian University of Munich, DE-80337 Munich, Germany; 6 Institute of Molecular Immunology and Experimental Oncology, Klinikum rechts der Isar, Technical University of Munich, DE-81675 Munich, Germany; 7 Department of Cardiothoracic Surgery, Jena University Hospital, DE-07747 Jena, Germany; 8 Present Address: School of Medicine, Division of Cardiology, Center for Metabolism and Mitochondrial Medicine, and Vascular Medicine Institute, University of Pittsburgh, Pittsburgh, PA 15261 USA

Correction to: *Gene Therapy* 10.1038/s41434-021-00235-z, published online 04 March 2021

We have become aware of an error in Supplementary Fig. 2 of this paper. Two loading controls for a western blot (ɑ-Tubulin and GAPDH) were immunolabeled on the same membrane and thus showed a similar expression pattern. Due to an unfortunate oversight, we cropped and presented ɑ-Tubulin twice. We here provide a corrected Supplementary Fig. 2b, replacing the erroneous panels with a new one from the original immunoblot, that now shows both ɑ-Tubulin (Cell Signaling, Cat. # 3873) and GAPDH (Cell Signaling, Cat. # 2118S) expression. Of note, the interpretation of the presented data is unchanged.

## Supplementary information

Supplemental Material

