# Supplementary material for: Alternative oxidase encoded by sequence-optimized and chemically-modified RNA transfected into mammalian cells is catalytically active
Source: Gene Ther. 2021 Mar 4;29(12):655–64. doi: 10.1038/s41434-021-00235-z (PMC9750868; doi:10.1038/s41434-021-00235-z)
Supplement: Supplementary file 1 — Supplemental Material [file 41434_2021_235_MOESM1_ESM.pdf]

Alternative oxidase encoded by sequence-optimized and chemically-modified RNA transfected into mammalian cells is catalytically active

Luca Giordano *et al.*,

Supplemental Material

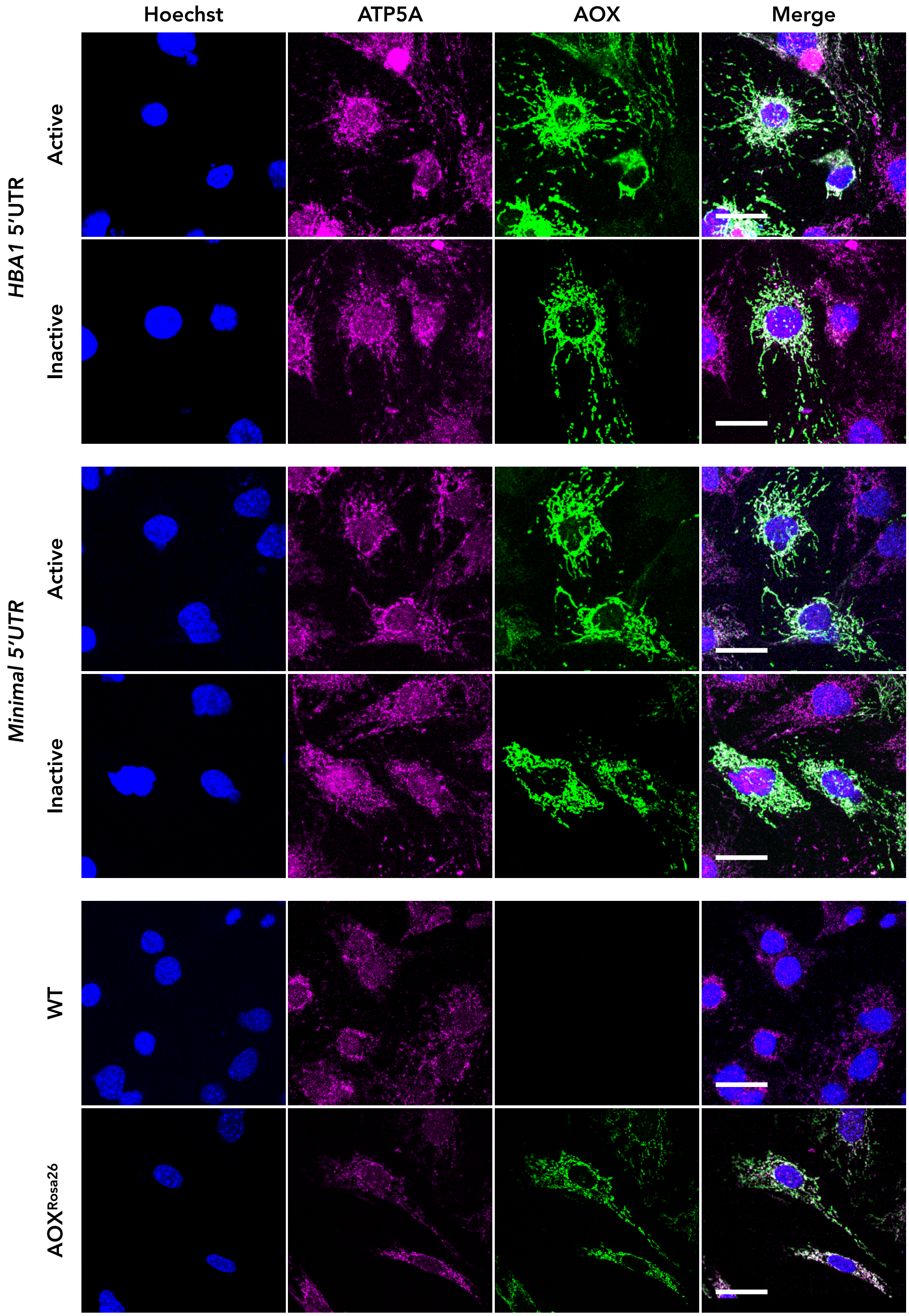

Supplementary fig. S1 Immunocytochemistry of iMEFs transfected with cmRNA variants

bearing the indicated 5′ UTR elements and encoding active or inactive AOX forms as shown. Non-transfected iMEFs derived from WT or AOX^{Rosa26}-transgenic mice were used as negative and positive controls, respectively. Hoechst, nuclear stain; ATP5A, mitochondrial positive control. Scale bars represent 30 μm.

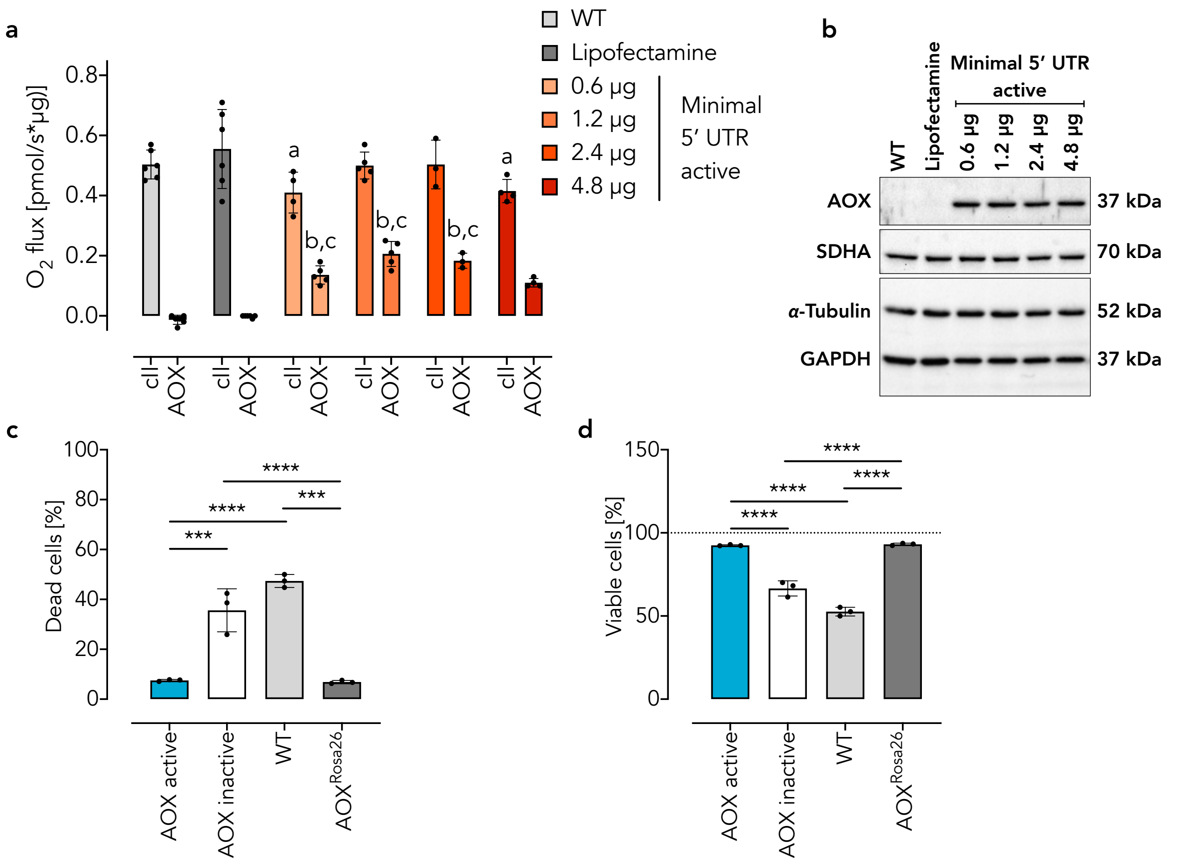

Supplementary fig. S2 Respiratory activity and protein expression in iMEFs transfected with increasing amounts of cmRNA encoding catalytically active AOX with the minimal 5´ UTR. (**a**) High-resolution respirometry using iMEFs 24 h after transfection with cmRNA amounts as indicated. WT, iMEFs from wild-type littermates of AOX transgenic mice; Lipofectamine, WT iMEFs mock-transfected using only lipofectamine; cII, oxygen consumption in the presence of succinate plus rotenone; AOX, oxygen consumption in the presence of succinate, rotenone and antimycin A after subtraction of residual oxygen consumption upon addition of n-propyl gallate (nPG). Data are shown as mean ± SD of n ≥ 3 independent experiments. a, $p \leq 0.05$ vs cII:Lipofectamine; b, $p \leq 0.05$ vs AOX:WT; c, $p \leq 0.05$ vs AOX:Lipofectamine calculated by two-way ANOVA and Tukey's multiple comparisons test. (**b**) Representative western blots of

iMEFs transfected with cmRNA amounts as indicated. SDHA, succinate dehydrogenase complex subunit A (mitochondrial loading control); α-Tubulin and GAPDH, glyceraldehyde 3-phosphate dehydrogenase (whole-cell loading control). Bar graphs show (**c**) total cell count and (**d**) dead cell count. AOX expressing iMEFs isolated from AOX^{Rosa26} were used as reference (100%) to normalize total cell number. Dead cells reported as ratio of dead cell number and total cell number expressed as percentage (%). Data shown as mean ± SD of n = 3 independent experiments with ***$p \leq 0.001$, ****$p \leq 0.0001$ calculated by one-way ANOVA and Tukey's multiple comparisons test.
